# A Novel TetR-Like Transcriptional Regulator Is Induced in Acid-Nitrosative Stress and Controls Expression of an Efflux Pump in Mycobacteria

**DOI:** 10.3389/fmicb.2017.02039

**Published:** 2017-10-23

**Authors:** Filomena Perrone, Barbara De Siena, Lidia Muscariello, Sharon L. Kendall, Simon J. Waddell, Margherita Sacco

**Affiliations:** ^1^Dipartimento di Scienze e Tecnologie Ambientali Biologiche e Farmaceutiche, Università della Campania “Luigi Vanvitelli”, Caserta, Italy; ^2^Department of Pathobiology and Population Science, Royal Veterinary College, London, United Kingdom; ^3^Wellcome Trust Brighton and Sussex Centre for Global Health Research, Brighton and Sussex Medical School, University of Sussex, Brighton, United Kingdom

**Keywords:** mycobacteria, TetR-family, transcription factors, binding motif, efflux pump, acid-nitrosative response, microarray analysis

## Abstract

*Mycobacterium tuberculosis* has the ability to survive inside macrophages under acid-nitrosative stress. *M. tuberculosis Rv1685c* and its ortholog in *M. smegmatis, MSMEG_3765*, are induced on exposure to acid-nitrosative stress. Both genes are annotated as TetR transcriptional regulators, a family of proteins that regulate a wide range of cellular activities, including multidrug resistance, carbon catabolism and virulence. Here, we demonstrate that *MSMEG_3765* is co-transcribed with the upstream genes *MSMEG_3762* and *MSMEG_3763*, encoding efflux pump components. RTq-PCR and GFP-reporter assays showed that the *MSMEG_3762/63/65* gene cluster, and the orthologous region in *M. tuberculosis* (*Rv1687c/86c/85c*), was up-regulated in a *MSMEG_3765* null mutant, suggesting that *MSMEG_3765* acts as a repressor, typical of this family of regulators. We further defined the *MSMEG_3765* regulon using genome-wide transcriptional profiling and used reporter assays to confirm that the *MSMEG_3762/63/65* promoter was induced under acid-nitrosative stress. A putative 36 bp regulatory motif was identified upstream of the gene clusters in both *M. smegmatis* and *M. tuberculosis* and purified recombinant MSMEG_3765 protein was found to bind to DNA fragments containing this motif from both *M. smegmatis* and *M. tuberculosis* upstream regulatory regions. These results suggest that the TetR repressor *MSMEG_3765/Rv1685c* controls expression of an efflux pump with an, as yet, undefined role in the mycobacterial response to acid-nitrosative stress.

## Introduction

Tuberculosis (TB) is still endemic in many low and middle-income countries and the high incidence of *Mycobacterium tuberculosis* multi-drug resistant strains continues to plague the control of TB worldwide (Ayabina et al., 2016; Malone and Gordon, 2016; Dheda et al., 2017). Indeed, *M. tuberculosis*, the causative agent of tuberculosis, is one of the most successful bacterial pathogens with approximately 10 million new infections and 2 million deaths per year; furthermore, it is estimated that one third of the world’s populations are latently infected. The success of *M. tuberculosis* is partly due to its ability to manipulate the intracellular environment of macrophages. Indeed, it is able to survive within macrophages by preventing phagosome maturation, antigen processing and apoptosis, and by creating a niche where bacteria remain metabolically active and capable of replication (Gan et al., 2008; Garton and O’Hare, 2013). Bacilli are able to grow in intra- and extra-cellular environments in spite of the presence of anti-microbial effectors such as reactive oxygen and nitric oxide intermediates, acidification or metal accumulation (Butler et al., 2010; Awuh and Flo, 2017). This is due to the low permeability of the mycobacterial cell envelope for many toxic molecules, to the ability of mycobacteria to detoxify reactive oxygen and reactive nitrogen molecules, and to the maintenance of a neutral intra-bacterial pH within acidic environments. Many of these mechanisms are still poorly understood (Ehrt and Schnappinger, 2009; Evangelopoulos et al., 2015). Model systems, including the fast-growing non-pathogenic *M. smegmatis*, have been widely used to highlight relevant aspects of *M. tuberculosis* physiology, exemplified by the modulation of gene expression by the DosR and Lsr2 regulators involved in dormancy and adaptation to hypoxic stress (Colangeli et al., 2009; Trauner et al., 2012; Agrawal et al., 2015).

In recent work, Cossu et al. (2013) profiled the responses of *M. tuberculosis* and *M. smegmatis* to acid-nitrosative multi-stress, simulating a macrophage-like environment. In these conditions, *Rv1685c* in *M. tuberculosis* and its ortholog *MSMEG_3765* in *M. smegmatis* were found to be up-regulated. Both genes are annotated as transcriptional regulators of the TetR family, sharing a high percentage of identity between their deduced amino acid sequences. Members of the TetR family of transcriptional regulators are widespread among bacteria (Cuthbertson and Nodwell, 2013). They control a wide range of cellular activities, like pathogenicity, osmotic stress responses and drug efflux (Balhana et al., 2015). The members of this family usually act as repressors, with an N-terminal DNA binding domain and a larger C-terminal domain that interacts with one or more ligands modulating the regulator’s ability to bind DNA. A diversity of ligands have been described, including antibiotics, bile acids and other toxic molecules, cell–cell signaling molecules, carbon sources, proteins and metal ions, underscoring a central role of the TetR-regulators in the adaptation of cellular processes to the changing environment (Cuthbertson and Nodwell, 2013).

Here, we characterize the TetR regulator *MSMEG_3765* (*Rv1685c*) using a combination of mutagenesis, local and global gene expression analyses, and DNA binding studies to show that it regulates the *MSMEG_3762/63/65* (and *Rv1687c/86c/85c*) operon encoding an efflux pump. This system is conserved in *M. tuberculosis* and our experiments suggest that this TetR regulator plays a novel role in the mycobacterial response to the intracellular environment.

## Materials and Methods

### Bacterial Strains and Culture Conditions

*Escherichia coli* TOP10 and DH5α were used as strains for cloning and *E. coli* BL21 (DE3) was used as a host for protein expression. *M. smegmatis* mc^2^155 and *M. tuberculosis* H37Rv were used throughout this work. The *E. coli* strains were grown in Luria-Bertani (LB) broth, while the mycobacterial strains were cultured in Middlebrook 7H9 broth (Difco) containing 10% oleic acid-albumin-dextrose-catalase supplement (Becton Dickinson) and 0.05% Tween 80. All strains were grown at 37°C with shaking. Hygromycin (200 μg ml^-1^ for *E. coli* and 100 μg ml^-1^ for *M. smegmatis*), kanamycin (50 μg ml^-1^ for *E. coli* and 25 μg ml^-1^ for *M. smegmatis*), 5-bromo-4-chloro-3-indolyl-β-D-galactopyranoside (Xgal 50 μg ml^-1^) and sucrose (2% w/v) were used for selection or screening as appropriate. Acid-nitrosative multi-stress was induced in 7H9 buffered medium at pH 5.3 by the addition of NaNO_2_ up to a final concentration of 5 mM for 5 h. Plasmids used throughout this work are listed in Supplementary Table S1. All primer sequences used in this study are listed in Supplementary Table S2.

### Bioinformatic Analysis

The bioinformatic analysis of the *MSMEG_3765* locus was conducted using BLAST and Clustal Omega. The analysis to identify putative TetR binding sites was conducted using MEME^1^ (Bailey et al., 2009). MEME was set to find palindromic motifs with a minimum width of 6 bp and a maximum of 50 bp. MEME was set to return a maximum of three motifs.

### Construction of the *M. smegmatis ΔMSMEG_3765* Mutant Strain

The *M. smegmatis ΔMSMEG_3765* mutant strain was isolated using a two-step homologous recombination strategy (Parish et al., 1999). A 804 bp fragment (up), containing the upstream flanking regions of *MSMEG_3765* (from 3829208 to 3829941), was PCR-amplified from *M. smegmatis* mc^2^155 genomic DNA using the forward upMS3765f and reverse upMS3765r primers, with HindIII-BamHI sites respectively, to clone the up fragment into p2NIL, yielding the pFP2 plasmid. A 906 bp fragment (dw), containing the downstream flanking regions of *MSMEG_3765* (from 3830395 to 3831300), was PCR-amplified from *M. smegmatis* mc^2^155 genomic DNA using the forward dwMS3765f and reverse dwMS3765r primers, with BamHI-PacI sites respectively, to clone the dw fragment into pFP2, yielding the pFP3 plasmid. To obtain the suicide delivery vector (pFP4), the PacI cassette from pGOAL19 was cloned into pFP3. pFP4 was electroporated into *M. smegmatis* mc^2^155 and single crossovers were selected using kanamycin, hygromycin and Xgal. A single blue kanamycin and hygromycin-resistant colony was streaked onto fresh media without selection, and incubated at 37°C for 3–5 days to allow for second recombination events, before selection on plates containing sucrose and Xgal. The white sucrose-resistant colonies were screened for kanamycin and hygromycin sensitivity, then analyzed by PCR to confirm the deletion in *MSMEG_3765*. The deletion event in *M. smegmatis ΔMSMEG_3765* was verified by sequencing.

### Construction of the *M. smegmatis ΔMSMEG_3765* Complemented Strain

To complement *M. smegmatis ΔMSMEG_3765*, a DNA fragment containing the 636 bp coding sequence of *MSMEG_3765* (including start and stop codons) was amplified from *M. smegmatis* mc^2^155 with forward cMS3765f and reverse cMS3765r primers. The forward primer included an optimized Shine–Dalgarno sequence (Andreu et al., 2010). The 636 bp fragment was cloned into the EcoRI-XhoI sites of pMV306hsp (Addgene#26155) to obtain pFP6, which was electroporated into *M. smegmatis ΔMSMEG_3765*. Recombinant clones were selected using kanamycin, and the insertion confirmed by PCR.

### RNA Extraction and Reverse Transcription Reactions

RNA for RT-PCR and RTq-PCR analyses were extracted from wild-type mc^2^155, knockout mutant and complemented strains from log-phase cultures under standard growth conditions. The bacteria were harvested, resuspended in 4 ml of RLT buffer (RNeasy kit – Qiagen), and vortexed using glass beads. Cell lysates were recovered by centrifugation and RNAs was extracted using RNeasy kit (Qiagen), according to the manufacturer’s instructions. RNA samples were treated with RQ1 DNase (Promega) for 30 min at 37°C, followed by heat inactivation. Finally, the quality and quantity of RNA was assessed using NanoDrop spectrophotometer analysis (Nanodrop, Thermo Scientific) and gel electrophoresis. Reverse transcription was performed for 15 min at 42°C in a total volume of 20 μl containing 1 μg total RNA (QuantiTect Reverse Transcription kit-Qiagen). As negative controls, samples without the reverse transcription step were used as template.

### RT-PCR and RT-qPCR Analyses

RT-PCR was performed to evaluate the transcriptional organization of the *MSMEG_3765* locus. Oligonucleotides were designed to span the intergenic regions from *MSMEG_3760* to *MSMEG_3765* (Supplementary Figure S1). As positive controls, each region was amplified using *M*. *smegmatis* mc^2^155 chromosomal DNA as template. Analysis by Real time PCR was performed using SYBR green technology on a 7500 RT-qPCR system instrument (Applied Biosystems). The *sigA* gene was used as an internal standard for expression analysis. The PCR conditions included an initial denaturation at 95°C for 10 min, followed by 40 cycles of amplification of 15 s at 95°C, 1 min at 60°C, and 30 s at 72°C. RT-qPCR analysis was performed in triplicate, and each assay included standard curves for both internal control and target genes, obtained by amplifying serial dilutions (ratio 1:10) of the samples. Relative expression levels were normalized using *sigA* and calculated using the 2^-ΔΔCt^ method (Livak and Schmittgen, 2001).

### Microarray Hybridization, and Data Analysis

Total RNA was extracted from mid-log phase *M. smegmatis* wt, *ΔMSMEG_3765* and complemented strains grown in 7H9/ADC at 37°C with shaking using the GTC/Trizol method (Waddell and Butcher, 2010). Samples were DNase-treated and purified using RNeasy columns (Qiagen), and the quality assessed by Agilent 2100 Bioanalyzer. Mycobacterial RNA (1 μg) was directly labeled with Cy3 fluorophore using the Universal Linkage System (ULS, Kreatech Diagnostics). Microarray hybridizations from three biological replicates (two for the complemented strain) were conducted as previously described (Tailleux et al., 2008) using an *M. smegmatis* microarray (Agilent Technologies) designed by the Bacterial Microarray Group at St. George’s (ArrayExpress accession number A-BUGS-39). To define the *ΔMSMEG_3765* regulon, significantly differentially expressed genes were identified comparing *ΔMSMEG_3765* to both wt and complemented strains using a moderated *t*-test (*p*-value < 0.05 with Benjamini and Hochberg multiple testing correction) and a >2.5-fold change. Fully annotated microarray data have been deposited in ArrayExpress (accession number E-MTAB-5869).

### GFP-Reporter Assay

To identify the region containing putative MSMEG_3765-responding operators, we cloned: (1) a 277 bp DNA fragment containing 43 bp coding sequence and 234 bp upstream from the ATG codon of *MSMEG_3760*, using MS13f and MS13r primers; (2) a 218 bp DNA fragment containing 106 bp of *MSMEG_3761* coding sequence, 36 bp of *MSMEG_3762* coding sequence and 76 bp of intergenic non-coding region, using MS14f and MS14r primers. The PCR products, obtained with iProof high-fidelity Taq (Bio-Rad), were cloned into the BamHI and ApaI sites of the *E. coli*–mycobacteria shuttle vector pFPV27 (Valdivia et al., 1996), yielding pFP13 and pFP14 plasmids, respectively. To analyze the promoter region of the *M. tuberculosis* H37Rv *Rv1687c* gene, we cloned a 200 bp DNA fragment, containing 40 bp coding sequence and 160 bp upstream from the ATG codon of *Rv1687c* into the pFPV27 plasmid, using Rv10f and Rv10r primers, yielding the pFP10 plasmid.

The resulting recombinant plasmids, together with the pFPV27 negative control plasmid (empty vector) and the pFPV27hsp positive control plasmid (carrying the mycobacterial hsp60 promoter) (Dhandayuthapani et al., 1995), were electroporated separately into *M. smegmatis* wt and *M. smegmatis ΔMSMEG_3765* cells. GFP fluorescence was measured as described previously (Carroll and James, 2009), with excitation at 490 nm and emission at 510 nm. To evaluate promoter activity of *MSMEG_3762* under acid-nitrosative multi-stress, *M. smegmatis* (pFP14) log-phase cells were incubated for 5 h in 7H9 buffered medium at pH 5.3 and supplemented with 5 mM NaNO_2_ before measuring fluorescence.

### Expression and Purification of Recombinant MSMEG_3765

The *MSMEG_3765* coding region (630 bp) was PCR-amplified from *M. smegmatis* mc^2^155 genomic DNA using eMS3765f and eMS3765r primers, and the product was cloned into the NdeI-XhoI sites of the pET-22b(+) expression vector. The recombinant plasmid, pFP5, was sequence verified and used to express and purify the C-terminally His-tagged TetR3765 protein. For expression, *E. coli* BL21 (DE3) cultures containing pFP5 were grown at 37°C to mid-exponential phase. Cultures were induced with 1 mM IPTG for 2 h at 37°C and harvested by centrifugation. The recombinant protein was purified using Profinity IMAC resins (Bio-Rad) following manufacturer’s instructions and concentrated using Centricon ultra-filtration spin columns (cut-off 10 kDa; Millipore) in 20 mM Tris HCl pH 8, 500 mM NaCl. The purified protein was correctly folded, as evaluated by circular dichroism spectroscopy (data not shown).

### Electrophoretic Mobility Shift Assays

Binding assays were performed incubating increasing concentrations of purified recombinant MSMEG_3765 (from 1.5 to 10 pmol) in HEPES 40 mM (pH 8.0), NaCl 150 mM, MgCl_2_ 20 mM, 10% glycerol with 0.5 pmol of *MSMEG_3762* (218 bp) or *Rv1687c* (200 bp) upstream fragments (used in the GFP assays). The binding reaction was performed at 37°C for 20 min. The resulting DNA/protein complexes were resolved on native PAGE gels (8% acrylamide:bisacrylamide 30:1) and stained with SYBR Safe DNA Gel Stain (Invitrogen). To probe the specificity of MSMEG_3765 binding activity to *MSMEG_3762* and *Rv1687c* upstream regions, reactions were incubated in the presence of 0.5 pmol *MSMEG_3760* upstream fragment (127 bp, primers mot3760f1/MS13r) as non-specific competitor DNA. The *MSMEG_3762* upstream fragment of 218 bp was refined into four shorter fragments for further EMSA analysis: (a) 133 bp containing 21 bp of the *MSMEG_3761* coding sequence, 36 bp of *MSMEG_3762* coding sequence and 76 bp of intergenic non-coding region (primers mot3762f1/MS14r); (b) 182 bp containing 106 bp of the *MSMEG_3761* coding sequence and 76 bp of intergenic non-coding region (primers MS14f/mot3762r1); (c) 97 bp containing 21 bp of the *MSMEG_3761* coding sequence and 76 bp of intergenic non-coding region (primers mot3762f1/mot3762r1); (d) 36 bp putative TetR binding motif.

## Results

### *MSMEG_3765* and the Surrounding Region Is Conserved in *M. tuberculosis* and Other Pathogenic Mycobacteria

**FIGURE 1:**
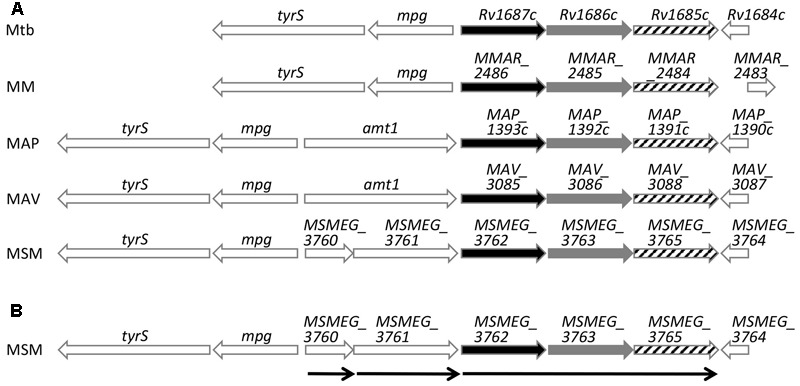
Schematic representation of *Rv1685c TetR* loci sequence alignments. **(A)** Alignment of *M. tuberculosis* H37Rv (Mtb), *M. marinum* (MM), *M. avium* subsp. *paratuberculosis* str. k10 (MAP), *M. avium* (MAV), and *M. smegmatis* (MSM); black bars indicate ABC transporter ATP-binding proteins, gray bars indicate ABC transporters and stripped bars indicate TetR transcriptional regulators. **(B)** Schematic representation of transcriptional units of the *M. smegmatis MSMEG_*3765 *tetR* locus determined by RT-PCR. Thin arrows indicate transcriptional units and direction of transcription.

*Mycobacterium tuberculosis* H37Rv *Rv1685c* and *M. smegmatis MSMEG_3765* TetR transcriptional regulators share 71% amino acid identity. In order to identify their distribution among other mycobacteria, we searched for orthologs in other species. The regulator is conserved in mycobacteria with amino acid identities ranging from 62 to 73% (**Figure 1A**). In all species, the regulator is preceded by two genes in the same transcriptional orientation, annotated as an ABC transporter ATP-binding protein and an ABC transporter, that also share high percentage of identity between species (68–79%).

### *MSMEG_3765* Is Co-transcribed with the Upstream Genes *MSMEG_3763* and *MSMEG_3762*

In order to define the transcriptional unit(s) in the *MSMEG_3765* locus, an RT-PCR analysis was performed on total RNA extracted from log phase *M. smegmatis* mc^2^155 using oligonucleotide pairs designed to detect transcripts of individual or co-transcribed genes. *MSMEG_3762, MSMEG_3763* and *MSMEG_3765* were found to be co-transcribed defining the *MSMEG_3762/63/65* operon (**Figure 1B** and Supplementary Figure S1). The analysis indicated that *MSMEG_3760* and *MSMEG_3761*, were not part of the 3 gene operon but were transcribed as monocistronic units (Supplementary Figure S1).

### MSMEG_3765 Acts as a Transcriptional Repressor of the *MSMEG_3762/63/65* Operon

**FIGURE 2:**
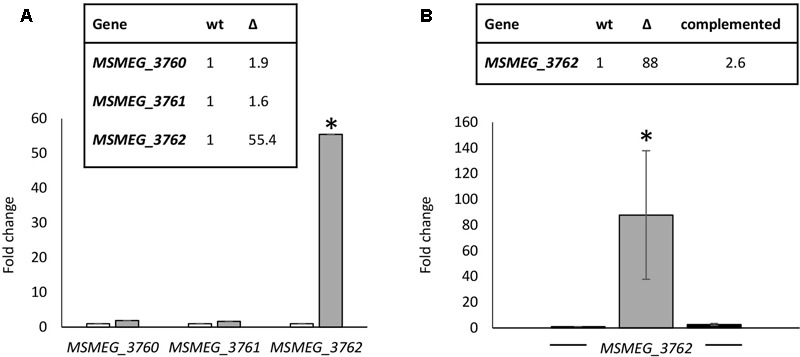
Comparative transcriptional analysis of the *M. smegmatis TetR* locus. **(A)** Expression levels of *MSMEG_3760, MSMEG_3761*, and *MSMEG_3762-63-65* were evaluated by RT-qPCR in wt (white) and *ΔMSMEG_*3765 (gray); **(B)** RT-qPCR analysis of the *MSMEG_3762-63-65* operon in wt (white), *ΔMSMEG_3765* (gray) and complemented *ΔMSMEG_3765* pFP6 (black) strains. Expression of each gene in the wild-type was arbitrarily assigned as 1. Error bars represent standard deviation of the mean values of three independent experiments and each RT-qPCR was carried out in triplicate (mean values are also shown in the boxes). *sigA* was used as reference gene and relative expression levels were calculated with the 2^-ΔΔCt^ method; ^∗^*p* < 0.001.

Many TetR-like proteins have been shown to regulate adjacent genes (Balhana et al., 2015). RTq-PCR analysis was used to determine the effect of deletion of *MSMEG_3765* on the expression of the surrounding genes. A *M. smegmatis* mutant strain carrying a 495 bp deletion in *MSMEG_3765* was generated, *ΔMSMEG_3765.* The deletion was also complemented using the integrative recombinant pFP6 plasmid, harboring the *MSMEG_3765* coding sequence under the control of the *hsp60* promoter. Deletion or complementation of *MSMEG_3765* did not affect growth in standard conditions, with a doubling time of ∼3 h for wt, mutant and complemented strains. Transcription of the *MSMEG_3762/63/65* operon (as assessed by measuring the first gene in the operon *MSMEG_3762*) was upregulated by 55-fold in the mutant strain versus the wt (**Figure 2A**), whereas the expression of *MSMEG_3760* and *MSMEG_3761* was unchanged. The expression of the *MSMEG_3762/63/65* operon was restored to wt levels in the complemented strain (**Figure 2B**). These data strongly suggest that MSMEG_3765 acts as a repressor of its own operon but does not control the expression of *MSMEG_3760* or *MSMEG_3761*.

Genome-wide transcriptional profiling was applied as an unbiased approach to further describe the *MSMEG_3765* regulon. The *MSMEG_3765* regulon was defined as genes significantly differentially expressed in *M. smegmatis ΔMSMEG_3765* compared to both wt and complemented strains to control for the possibility of polar effects of gene manipulation. Microarray analysis confirmed that *MSMEG_3765* acts as a repressor of *MSMEG_3762* and *MSMEG_3763* with both genes induced in the *ΔMSMEG_3765* genetic background (**Table 1**).

**Table 1:** The TetR-MSMEG_3765 regulon determined by genome-wide transcriptional profiling.

Gene name	*ΔMSMEG_3765* vs. wt Fold induction	*ΔMSMEG_3765* vs. wt Adjusted *p*-value	*ΔMSMEG_3765* vs. comp Fold induction	*ΔMSMEG_3765* vs. comp Adjusted *p*-value
*MSMEG_3762*	19	4.7 × 10^-5^	18	0.013
*MSMEG_3763*	6	4.9 × 10^-3^	4	0.036

*Genes significantly differentially expressed in *ΔMSMEG_3765* compared to both wt and complemented strains are listed, defining the MSMEG_3765 regulon. Fold change in the *ΔMSMEG_3765* relative to wt and complemented genetic backgrounds are detailed, alongside the *p*-values from moderated *t*-tests after multiple testing correction.*

### Identification of a Regulatory Motif Upstream of the *MSMEG_3762/63/65* Operon

**FIGURE 3:**
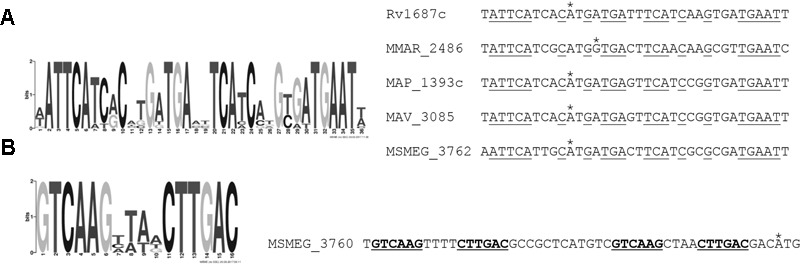
MEME motif analysis for putative TetR binding sites. **(A)** Palindromic motif (36 bp) identified in the intergenic region of *M. tuberculosis Rv1687c, M. marinum MMAR_2486, M. avium* subsp. *paratuberculosis MAP_1393c, M. avium MAV_3085* and *M. smegmatis MSMEG_3762* genes, extending into the predicted coding regions (*e*-value = 3.1 × 10^-27^). **(B)** Palindromic motifs identified in the *MSMEG_3760* upstream region, located –47 bp (*e*-value = 7.53 × 10^-10^) and –19 bp (*e*-value = 2.06 × 10^-9^) upstream of the ATG start codon. Asterisks indicate the predicted start codon; motifs identified by MEME are underlined.

TetR proteins often bind palindromic regulatory sequences, therefore we searched for palindromic motifs upstream of the *MSMEG_3762/63/65* operon. A conserved 36 bp region, containing a 34 bp palindrome, at the 5′ intergenic region extending into the coding sequence of *M. tuberculosis Rv1687c, M. smegmatis MSMEG_3762, M. marinum MMAR_2486, M. avium* subsp. *paratuberculosis MAP_1393c, M. avium MAV_3085* genes was found (**Figure 3A**). This motif overlaps with a putative TATA box found 10 bp upstream of the translation start site for each gene.

TetR proteins often control divergently oriented genes, therefore we searched for additional motifs in the intergenic region upstream of *mpg/amt1* in *M. avium paratuberculosis* and *M. avium* and *mpg/MSMEG_3760* in *M. smegmatis.* Two copies of a 16 bp motif containing a 6 bp palindrome were found in this region in the *M. smegmatis* genome but not in the other genomes (**Figure 3B**). The RTq-PCR data suggested that this motif was not recognized by *MSMEG_3765*, however, reporter assays were performed to determine the functionality of both motifs.

### Characterization of MSMEG_3765 Binding Sites Using GFP-Promoter Probes

**FIGURE 4:**
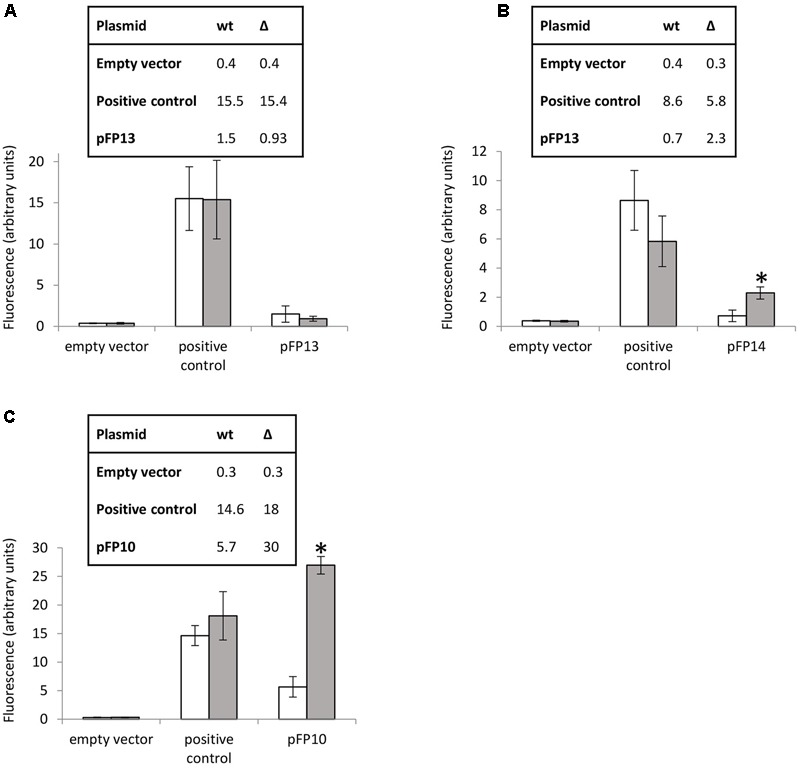
MSMEG_3765 binding upstream of *MSMEG_3762* and *Rv1685c* established by promoter activity. Promoter probe assays of **(A)** *MSMEG_3760* upstream region (pFP13); **(B)** *MSMEG_3762* upstream region (pFP14); and **(C)** *Rv1687c* upstream region (pFP10). Empty vector (pFPV27) acted as the negative control; pFPV27hsp, carrying the mycobacterial hsp60 promoter, acted as the positive control. The analysis was performed in *M. smegmatis* wt (white) and *ΔMSMEG_3765* (gray) genetic backgrounds. All the experiments were performed three times in triplicate. Error bars represent standard deviation of the mean (mean values are also shown in the boxes); ^∗^*p* < 0.0001.

Several reporter strains were made by cloning the promoter regions (including the motif regions) from *MSMEG_3760, MSMEG_3762/63/65*, and *Rv1687c/85c/85c* upstream of GFP to assay promoter activity. Promoterless GFP (empty vector) and GFP under the control of the *hsp60* promoter were used as negative and positive controls, respectively. Expression of GFP from the *MSMEG_3760* promoter resulted in a small but significant increase in fluorescence compared to empty vector (*p* < 0.01), but no significant difference was observed between wt and *ΔMSMEG_3765* backgrounds (**Figure 4A**). Conversely, expression of GFP from the *MSMEG_3762/63/65* promoter showed a significant increase (3.2-fold, *p* < 0.0001) in the *ΔMSMEG_3765* compared to wt (**Figure 4B**). These results suggest that the promoter region upstream of *MSMEG_3760* is active but not controlled by MSMEG_3765, while the *MSMEG_3762/63/65* promoter is active and negatively regulated by MSMEG_3765. Since the motif identified in this region was also present in the *M. tuberculosis Rv1687c/86c/85c* gene cluster, a 200 bp fragment, containing the *Rv1687c* upstream sequence was assayed in *M. smegmatis* wt and *ΔMSMEG_3765* strains. Promoter activity was detected in both genetic backgrounds with a fivefold increase (*p* < 0.0001) in expression in the mutant strain (**Figure 4C**), suggesting that the regulation of this gene cluster is conserved in *M. tuberculosis*.

**FIGURE 5:**
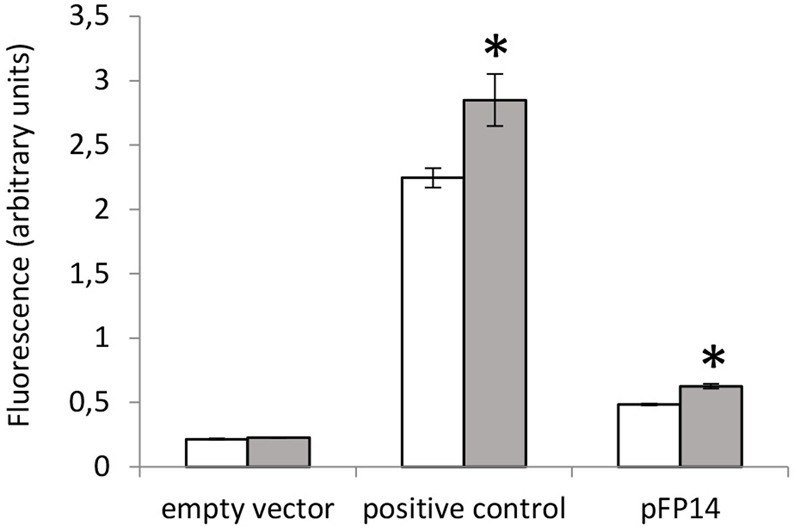
Acid-nitrosative stress induced-activity at the *MSMEG_3762* upstream region by promoter probe assay. GFP assay of the *MSMEG_3762* upstream region (pFP14) in wt *M. smegmatis* in standard conditions (white) and after acid-nitrosative stress (gray). Empty vector (pFPV27) acted as the negative control; pFPV27hsp, carrying the mycobacterial hsp60 promoter, acted as the positive control. The experiment was performed twice in triplicate. Error bars represent standard deviation of the mean; ^∗^*p* < 0.01.

Previous reports indicated that the *Rv1687c/86c/85c* gene cluster is induced by acid-nitrosative stress (Cossu et al., 2013). Therefore, we measured the promoter activity of the *MSMEG_3762/63/65* upstream region under the same conditions. A small but significant increase in promoter activity was detected after exposure to 5 mM NaNO_2_ at pH 5.3 (**Figure 5**), further evidence that this TetR-regulator is involved in the response to acid-nitrosative stress in *M. smegmatis* and *M. tuberculosis*.

### Confirmation of MSMEG_3765 DNA Binding and Target Sequence by EMSA

The promoter studies suggested that MSMEG_3765 binds to the motif upstream of the *MSMEG_3762/63/65* cluster but not to the motif identified upstream of *MSMEG_3760.* To verify the binding activity of MSMEG_3765, we conducted an electrophoretic mobility shift assay (EMSA) with the purified recombinant protein. A preliminary experiment using the three DNA fragments upstream of the *MSMEG_3760, MSMEG_3762/63/65*, and *Rv1687c86c/85c* genes (from the GFP assays) was performed. The upstream *MSMEG_3760* fragment did not show a shift of electrophoretic mobility in the presence of the MSMEG_3765 recombinant protein, while the other two DNA fragments were shifted in the presence of the protein (Supplementary Figure S2).

**FIGURE 6:**
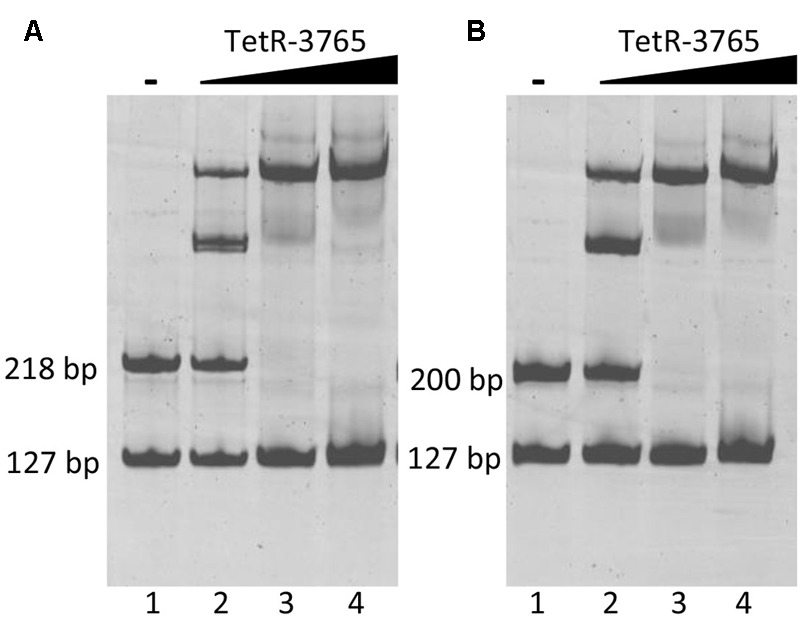
Binding of TetR-MSMEG_3765 to *MSMEG_3762* and *Rv1687c* promoters verified by EMSA. **(A)** *M. smegmatis MSMEG_3762* upstream fragment (218 bp); **(B)** *M. tuberculosis Rv1687c* upstream fragment (200 bp). Lane 1: negative control in which no protein was added. Lanes 2–4: binding reactions in the presence of 1.5, 5, 10 pmol of recombinant MSMEG_3765 showing a concentration-dependent gel shift in **(A,B)**. All reactions were carried out in the presence of the *MSMEG_3760* upstream fragment (127 bp) as the negative control fragment.

Electrophoretic mobility shift assay experiments were then performed using the 127 bp upstream *MSMEG_3760* fragment as a negative control in co-migration experiments either with *MSMEG_3762* or *Rv1687c* upstream sequences. Increasing input of recombinant MSMEG_3765 protein resulted in enhanced shift of the *MSMEG_3762* upstream fragment (218 bp) up to saturation (**Figure 6A**, lanes 2–4), while no shift was detected for the negative control fragment. In addition, a MSMEG_3765 concentration-dependent shift in electrophoretic mobility was observed for the *Rv1687c* upstream fragment (200 bp), also up to saturation with the highest concentration of MSMEG_3765 tested (**Figure 6B**, lanes 2–4). The EMSA analysis shows specific binding activity of MSMEG_3765 at the upstream regulatory regions of the *M. smegmatis MSMEG_3762/63/65* and homologous *M. tuberculosis Rv1687c/86c/85c* operons.

**FIGURE 7:**
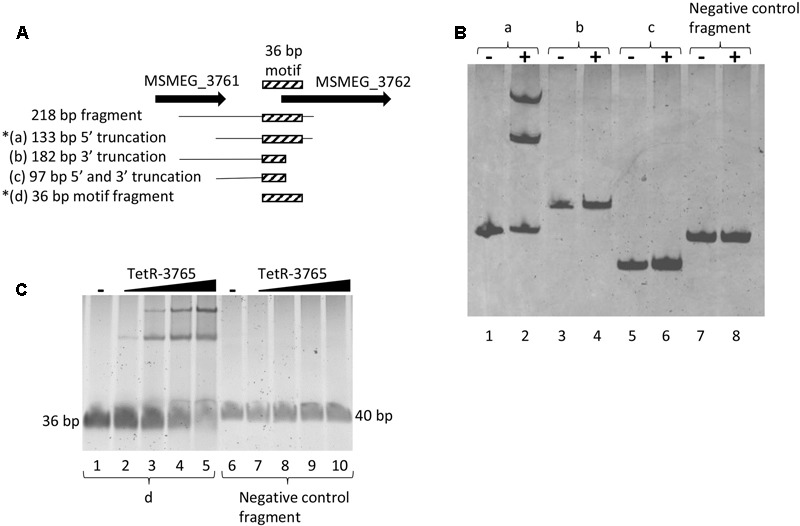
Refinement of the MSMEG_3765 binding site to 133 bp *MSMEG_3762* upstream sequence. **(A)** Schematic representation of DNA fragments used in the EMSA assay; the hatched box indicates the position of the conserved 36 bp TetR-binding motif (**Figure 3A**) truncated in (b) and in (c). Asterisks mark MSMEG_3765-binding by EMSA. **(B)** EMSA assay using the (a), (b) and (c) fragments and a 127 bp fragment containing the *MSMEG_3760* promoter as negative control (Negative control fragment). Five pmol of the recombinant MSMEG_3765 protein was used in the binding reactions (+), while no protein was added in the controls (–). **(C)** EMSA assay using the d fragment (lanes 1–5) and a 40 bp *MSMEG_3760* upstream fragment (Negative control fragment, lanes 6–10) containing the palindromic motif shown in **Figure 3B**. Lanes 1 and 6: no protein was added (–). Lanes 2–5 and 7–10: binding reactions in the presence of 1.5, 5, 10, and 20 pmol recombinant MSMEG_3765.

The 218 bp *MSMEG_3762* upstream fragment was truncated for further EMSA. Three DNA fragments were analyzed: (a) a 133 bp DNA fragment depleted of 85 bp *MSMEG_3761* coding sequence (truncated at the 5′ end); (b) a 182 bp DNA fragment depleted of 36 bp *MSMEG_3762* coding sequence (truncated at the 3′ end); (c) a 97 bp DNA fragment depleted of 85 bp *MSMEG_3761* coding sequence and of 36 bp *MSMEG_3762* coding sequence (truncated at the 5′ and 3′ ends) (**Figure 7A**). Only the 133 bp fragment, extending up to the 12th codon into the *MSMEG_3762* coding region, retained binding activity for the MSMEG_3765 protein (**Figure 7B**), showing that truncation of the motif at the 3′ end destroyed binding.

To further characterize the DNA motif for MSMEG_3765 binding, the 36 bp *MSMEG_3762* upstream fragment (**Figure 3A**) and a 40 bp *MSMEG_3760* upstream fragment, containing the palindromic motif shown in **Figure 3B**, were tested by EMSA. The former fragment showed a MSMEG_3765 concentration-dependent shift in electrophoretic mobility, while the latter fragment was not affected by the presence of the protein (**Figure 7C**). These data correlate with the MEME analysis (**Figure 3A**) and demonstrate that MSMEG_3765, acting as transcriptional repressor, binds to a 36 bp TetR-like motif extending into the coding sequence of *MSMEG_3762* and *M. tuberculosis* ortholog *Rv1687c* operons, controlling expression.

## Discussion

*Mycobacterium tuberculosis* is a well-adapted intracellular pathogen, employing multiple strategies to survive within macrophages. Many of the mechanisms that enable *M. tuberculosis* to survive stresses encountered in macrophages are still poorly understood. Experimental strategies involving *in vitro*-simulated phagosomal environments have been widely used to highlight differential gene expression of *M. tuberculosis* to the changing microenvironment (Kendall et al., 2004; Bacon and Marsh, 2007; Voskuil et al., 2011; Green et al., 2014; Salina et al., 2014; Namouchi et al., 2016; Bansal et al., 2017; Botella et al., 2017). In a comparative study, *M. tuberculosis* and *M. smegmatis* responses to acid nitrosative stress, mimicking the macrophage environment, were determined by transcriptional profiling (Cossu et al., 2013). *Rv1685c* in *M. tuberculosis* and its ortholog *MSMEG_3765* in *M. smegmatis*, both annotated as TetR transcriptional regulators, were found to be up-regulated. Members of the TetR family of transcriptional regulators are very common in bacteria and are involved in the control of efflux pumps, along with other cellular activities (Cuthbertson and Nodwell, 2013; Deng et al., 2013; Xu, 2016).

In this study, we show that *MSMEG_3765* is co-transcribed with *MSMEG_3762* and *MSMEG_3763* (encoding an ABC transporter system), and that the *MSMEG_3762/63/65* operon is regulated by MSMEG_3765. Given that this region and regulatory motif is conserved in *M. tuberculosis*, it is likely that the equivalent regulator in *M. tuberculosis* (*Rv1685c*) also controls the orthologous region in *M. tuberculosis* (*Rv1687c/86c/85c*). This is supported by the observation that the *Rv1687c* promoter is de-repressed in *ΔMSMEG_3765* genetic background (**Figure 4C**). Understanding the function of *MSMEG_3762/63/65* and *Rv1687c/86c/85c* is crucial to identifying the physiological role of this tightly regulated system.

In addition to up-regulation by acid-nitrosative stress, *Rv1687c/86c/85c* have been shown to be induced upon exposure to triclosan and lupulone, compounds that show potential as anti-mycobacterial agents, therefore it is feasible to speculate that the TetR-regulated ABC transporter is involved in drug efflux (Betts et al., 2003; Wei et al., 2014). A recent study, however, has shown that over-expression of *Rv1686c-Rv1687c* did not result in an increase in MIC to these compounds and further work is needed to investigate the role of this cluster in drug efflux (Gomez et al., 2016). The observation that other mycobacterial ABC transporters are involved in drug resistance in clinical isolates highlights the importance of extending our knowledge of the function and regulation of this cluster (Hao et al., 2011; Wang et al., 2013). Given the observation that the ABC transporter is up-regulated in acid-nitrosative stress, and in *M. tuberculosis* after macrophage infection (Schnappinger et al., 2003; Rohde et al., 2007) it is tempting to speculate that over-expression of the transporter may be functionally significant in the export of toxic compounds generated under these conditions, although ABC transporters may have multiple other roles in virulence including secretion of enzymes and siderophores, and the import of nutrients, ions and osmoprotective moieties (Lewis et al., 2012).

Of the 52 TetR regulators in the *M. tuberculosis* genome only a small percentage have been characterized (Balhana et al., 2015). In this study we have predicted and verified a novel regulatory motif, this is a 36 bp imperfect palindrome at the 5′ intergenic region extending into the coding sequence of *M. tuberculosis Rv1687c, M. marinum MMAR_2486, M. avium* subsp. *paratuberculosis MAP_1393c, M. avium MAV_3085*, and *M. smegmatis MSMEG_3762* genes (**Figure 3**). Short (16 bp) palindromic motifs are frequently described for this family of regulators, although there are exceptions. EthR, a TetR regulator involved in ethionamide bio-activation binds to a 55 bp operator containing imperfect direct repeats (Engohang-Ndong et al., 2004). We have unequivocally demonstrated binding of purified recombinant MSMEG_3765 to a short 36 bp fragment containing this motif and shown that this binding is abrogated when the motif is truncated at the 3′ end. Further work is necessary in order to determine the affinity of the regulator for the motif and the ligand of this tightly regulated system involved in the mycobacterial response to antimicrobial drugs and the macrophage intracellular environment.

## Author Contributions

FP, BDS, and LM performed the experimental work under the supervision of MS, and FP performed the microarrays and GFP assays under the supervision of SW, at the University of Sussex. SK and FP performed the bioinformatics work. MS and SK wrote the manuscript with a consistent contribution of FP, LM, and SW.

## Conflict of Interest Statement

The authors declare that the research was conducted in the absence of any commercial or financial relationships that could be construed as a potential conflict of interest.

## Footnotes

1 http://meme-suite.org/
