# Supplementary material for: A Novel TetR-Like Transcriptional Regulator Is Induced in Acid-Nitrosative Stress and Controls Expression of an Efflux Pump in Mycobacteria
Source: Front Microbiol. 2017 Oct 23;8:2039. doi: 10.3389/fmicb.2017.02039 (PMC5660060; doi:10.3389/fmicb.2017.02039)
Supplement: Supplementary file 1 [file Table_1.PDF]

Table S1. Plasmid used in this work

Name	Applications	Description	Reference
p2NIL	*M. smegmatis ΔMSMEG_3765* isolation	High copy suicide vector with Kanamycin resistance	Parish *et al*., 1999
pFP2	*M. smegmatis ΔMSMEG_3765* isolation	p2NIL+*MSMEG_3765* upstream region	This work
pFP3	*M. smegmatis ΔMSMEG_3765* isolation	pFP2+*MSMEG_3765* downstream region	This work
pGOAL19	*M. smegmatis ΔMSMEG_3765* isolation	Marker Cassette conferring ampicillin resistance, sucrose sensibility and carrying the *LacZ* gene	Parish *et al*., 1999
pFP4	*M. smegmatis ΔMSMEG_3765* isolation	pFP3+pGOAL19 marker cassette	This work
pMV306hsp	*M. smegmatis ΔMSMEG_3765* complementation	Mycobacteria integrating vector with hsp60 promoter	Andreu *et al*., 2010
pFP6	*M. smegmatis ΔMSMEG_3765* complementation	pMV306hsp+*MSMEG_3765* coding sequence	This work
pFPV27	*M. smegmatis* GFP assay	Mycobacteria expression	Valvidia *et al*., 1996
pFPV27hsp	*M. smegmatis* GFP assay	Mycobacteria expression under the hsp60 promoter	Dhandayuthapani *et al*., 1995
pFP13	*M. smegmatis* GFP assay	pFPV27+*MSMEG_3760* upstream region	This work
pFP14	*M. smegmatis* GFP assay	pFPV27+*MSMEG_3762* upstream region	This work
pFP10	*M. tuberculosis* GFP assay	pFPV27+intergenic region between *mpg* and *Rv1687c*	This work
pET-22b(+)	TetR3765 expression	Bacterial expression with C-terminal His tag	Novagen
pFP5	TetR3765 expression	pet22b+ *MSMEG_3765* coding sequence	This work
