# Supplementary material for: A Novel TetR-Like Transcriptional Regulator Is Induced in Acid-Nitrosative Stress and Controls Expression of an Efflux Pump in Mycobacteria
Source: Front Microbiol. 2017 Oct 23;8:2039. doi: 10.3389/fmicb.2017.02039 (PMC5660060; doi:10.3389/fmicb.2017.02039)
Supplement: Supplementary file 2 [file Table_2.PDF]

Table S2. Oligos used in this work

**Construction of the *M. smegmatis ΔMSMEG_3765* mutant strain**		
**Primer name**	**Sequence**	**Description**
upMS3765f	5'-CCCAAGCTTCGACCACCGCAGCGTCGCGATG-3'	Forward primer for the amplification of upstream *MSMEG_3765* fragment
upMS3765r	5'-CGGGATCCGGTGTCAGAGGTGGCGGTCAACTC-3'	Reverse primer for the amplification of upstream *MSMEG_3765* fragment
dwMS3765f	5'-CGGGATCCCAGTACGTGGCATCGCAG-3'	Forward primer for the amplification of downstream *MSMEG_3765* fragment
dwMS3765r	5'-CCTTAATTAACTGACTGGAGGATTGATGGCAGC-3'	Reverse primer for the amplification of downstream *MSMEG_3765* fragment
**Construction of the *M. smegmatis ΔMSMEG_3765* complemented strain**		
**Primer name**	**Sequence**	**Description**
cMS3765f	5'GGAATTCAGAAGGAGAAGTACCGATGACCGCCACCTCTGAC-3'	Forward primer for the amplification of *MSMEG_3765* gene
cMS3765r	5'-CCGCTCGAGCTAATCGAGCGCGCCGAATCC-3'	Reverse primer for the amplification of *MSMEG_3765* gene
**RT-PCR and RT-qPCR anal**		
**Primer name**	**Sequence**	**Description**
MS3760f	5'-GACGAACCGACCACCGAT-3'	Forward primer for *MSMEG_3760* and *MSMEG_3760*/*MSMEG_3761* cotranscription
MS3760r	5'-CCTCCGGTTGTACTTCTGGT-3'	Reverse primer for *MSMEG_3760*
MS3761f	5'-CGCACAAGGACTCTGTCATC-3'	Forward primer for *MSMEG_3761* and *MSMEG_3761*/*MSMEG_3762* cotranscription
MS3761r	5'-ACTCCAGCAGGCCGATAAT-3'	Reverse primer for *MSMEG_3761* and *MSMEG_3760*/*MSMEG_3761* cotranscription
MS3762f	5'-GATCTGTGGCAGCAGTTCAA-3'	Forward primer for *MSMEG_3762*/*MSMEG_3763* cotranscription
MS3762r	5'-GGAACTGCATGAGGTGTCCT-3'	Reverse primer for *MSMEG_3761*/*MSMEG_3762*

		cotranscription
MS3763f	5'-GCTGATCGTGATGTTCCTGA-3'	Forward primer for *MSMEG_3763*/*MSMEG_3765* cotranscription
MS3763r	5'-GTATGGAACCCCAGCAACC-3	Reverse primer for *MSMEG_3762*/*MSMEG_3763* cotranscription
MS3765r	5'-GAGTTCGGAATCCCACAGC-3'	Forward primer for *MSMEG_3763*/*MSMEG_3765* cotranscription
MS2758f	5'-CCAAGGGCTACAAGTTCTCG-3'	Forward primer for *MSMEG_2758* (*sigA*)
MS2758r	5'-CTTGTTGATCACCTCGACCA-3'	Reverse primer for *MSMEG_2758 (sigA)*
**GFP-reporter and EMSA assay**		
**Primer name**	**Sequence**	**Description**
MS13f	5'-CGGGATCCGGAACGAATGCGACGCCGCG-3'	Forward primer for the amplification of the *MSMEG_3760* upstream region
MS13r	5'-CGGGCCCGATCCACGCTGGCCATGGCAT C-3'	Reverse primer for the amplification of the *MSMEG_3760* upstream region
MS14f	5'-CGGGATCCGATCCTGCGGGGCGCCGAC G-3'	Forward primer for the amplification of the *MSMEG_3762* upstream region
MS14r	5'-CGGGCCCCGTCGCTGACAATTCATCGC-3'	Reverse primer for the amplification of the *MSMEG_3762* upstream region
Rv10f	5'-CGGGCCCCACCGTCGCGAAGTAATT-3'	Forward primer for the amplification of the *Rv1687c* upstream region
Rv10r	5'-CCGGATCCGACCTCGACCACCATCGC-3'	Reverse primer for the amplification of the *Rv1687c* upstream region
mot3760f1	5'-GACGCTCATCGATGCGATTGTGC -3'	Forward primer for the amplification of the MSMEG_3760 upstream region
mot3762f1	5'- CTGCTGGAGTCCGCCGCCTG-3'	Forward primer for the amplification of the MSMEG_3762 upstream region

mot3762r1	5’-GCAATGAATTCTAGGCCTGCCGTCG -3’	Reverse primer for the amplification of the MSMEG_3762 upstream region
**Expression and purification of recombinant MSMEG_3765**		
**Primer name**	**Sequence**	**Description**
eMS3765f	5’-GGAATTCCATATGACCGCCACCTCTGACACCGG-3’	Forward primer for the amplification of the *MSMEG_3765* gene
eMS3765r	5’-CCGCTCGAGATCGAGCGCGCCGAATCC-3	Reverse primer for the amplification of the *MSMEG_3765* gene
