# Supplementary figures and images for: A Novel TetR-Like Transcriptional Regulator Is Induced in Acid-Nitrosative Stress and Controls Expression of an Efflux Pump in Mycobacteria
Source: Front Microbiol. 2017 Oct 23;8:2039. doi: 10.3389/fmicb.2017.02039 (PMC5660060; doi:10.3389/fmicb.2017.02039)

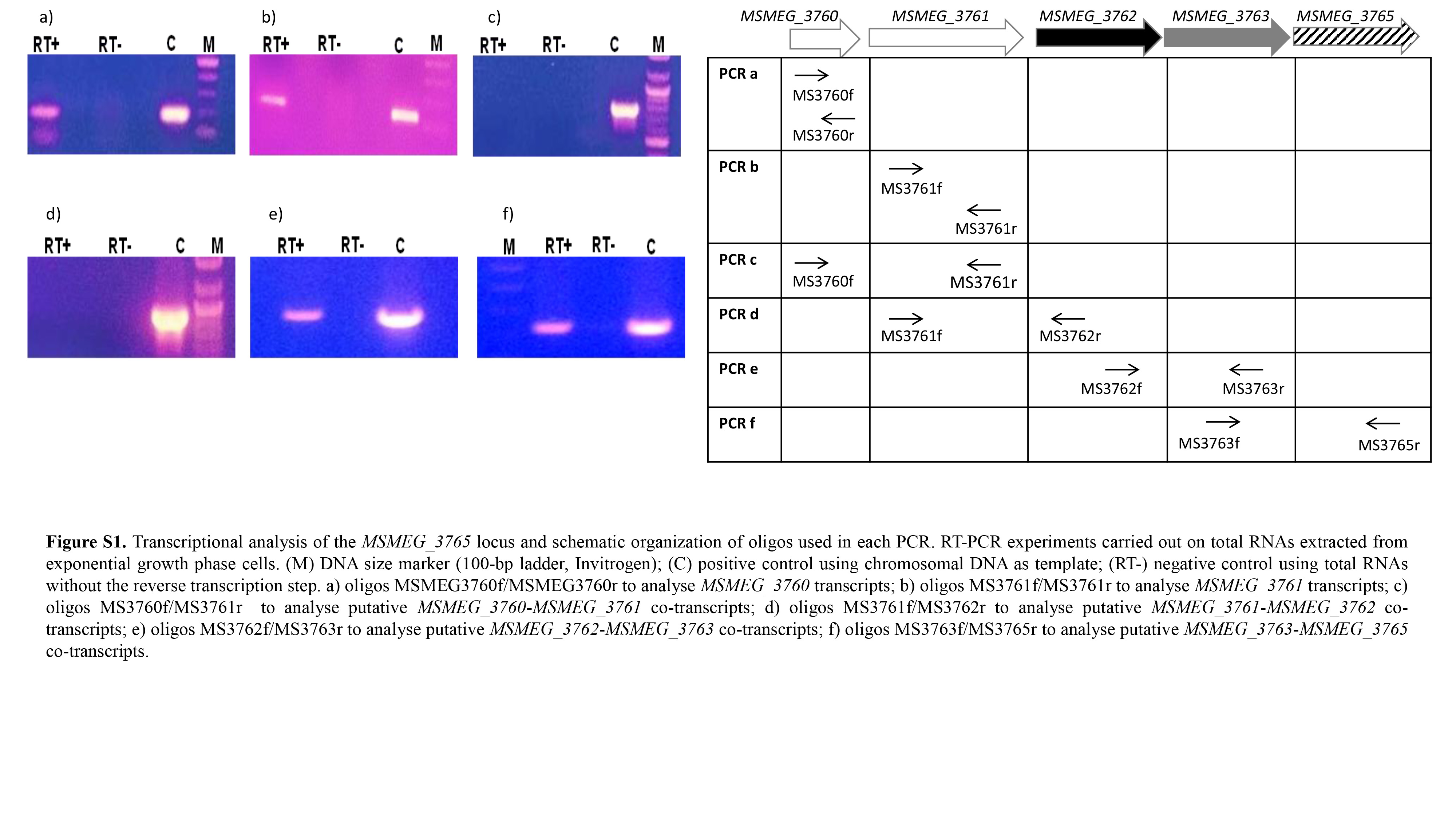

Supplement: Supplementary file 3 [file Image_1.jpg]

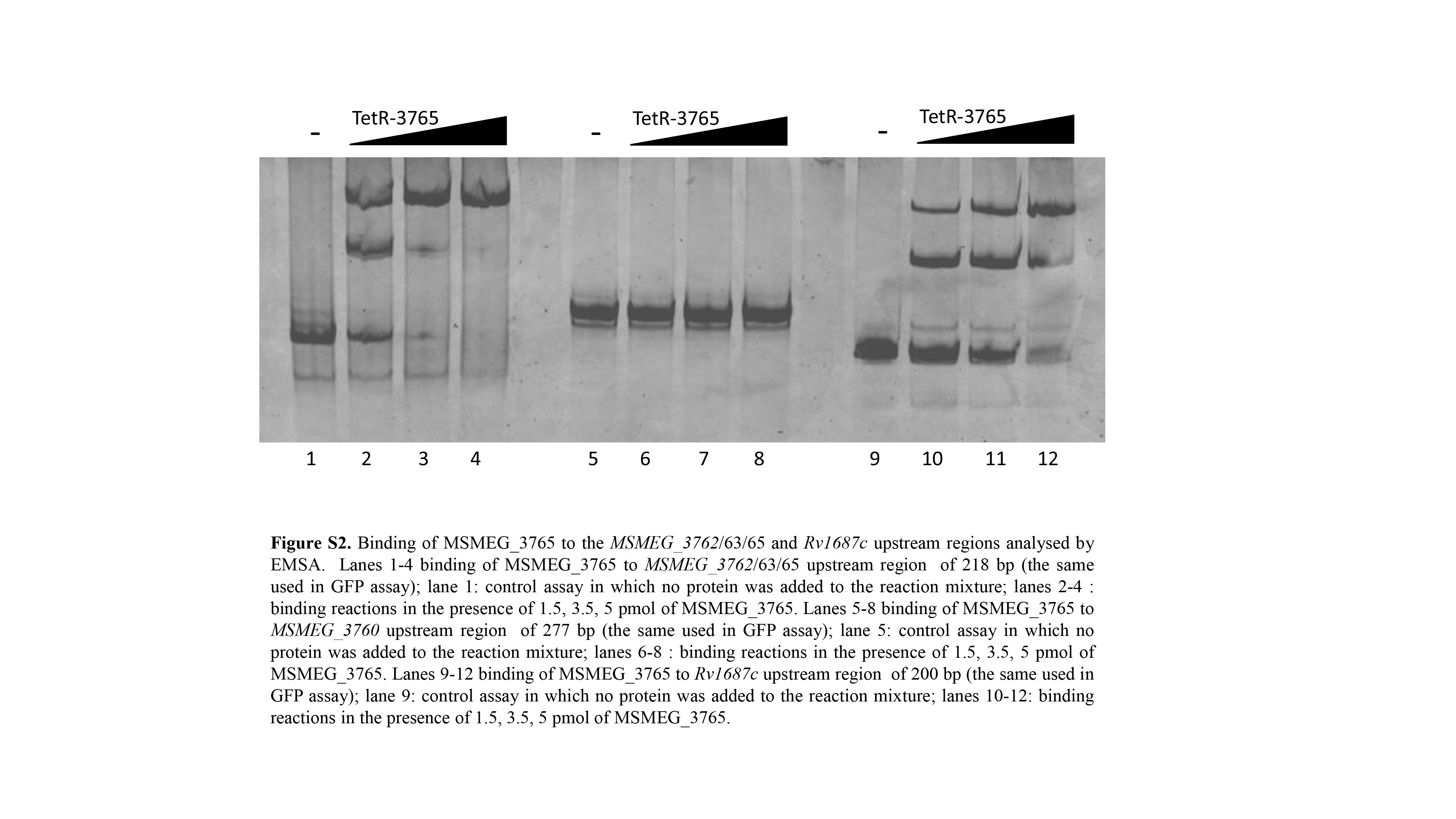

Supplement: Supplementary file 4 [file Image_2.jpg]
